# A Microfluidic Diagnostic Device Capable of Autonomous Sample Mixing and Dispensing for the Simultaneous Genetic Detection of Multiple Plant Viruses

**DOI:** 10.3390/mi11060540

**Published:** 2020-05-26

**Authors:** Daigo Natsuhara, Keisuke Takishita, Kisuke Tanaka, Azusa Kage, Ryoji Suzuki, Yuko Mizukami, Norikuni Saka, Moeto Nagai, Takayuki Shibata

**Affiliations:** 1 Department of Mechanical Engineering, Toyohashi University of Technology, Toyohashi, Aichi 441-8560, Japan; k-takishita@mems.me.tut.ac.jp (K.T.); k-tanaka@mems.me.tut.ac.jp (K.T.); kage@me.tut.ac.jp (A.K.); nagai@me.tut.ac.jp (M.N.); 2 Agro-Environmental Division, Aichi Agricultural Research Center, Nagakute, Aichi 480-1193, Japan; riyouji_3_suzuki@pref.aichi.lg.jp (R.S.); yuuko_mizukami@pref.aichi.lg.jp (Y.M.); norikuni_saka@pref.aichi.lg.jp (N.S.)

**Keywords:** multiplex genetic diagnosis, viral infectious diseases, plant viruses, loop-mediated isothermal amplification (LAMP), autonomous sample dispensing, microfluidic device, micro TAS

## Abstract

As an efficient approach to risk management in agriculture, the elimination of losses due to plant diseases and insect pests is one of the most important and urgent technological challenges for improving the crop yield. Therefore, we have developed a polydimethylsiloxane (PDMS)-based microfluidic device for the multiplex genetic diagnosis of plant diseases and pests. It offers unique features, such as rapid detection, portability, simplicity, and the low-cost genetic diagnosis of a wide variety of plant viruses. In this study, to realize such a diagnostic device, we developed a method for the autonomous dispensing of fluid into a microchamber array, which was integrated with a set of three passive stop valves with different burst pressures (referred to as phaseguides) to facilitate precise fluid handling. Additionally, we estimated the mixing efficiencies of several types of passive mixers (referred to as chaotic mixers), which were integrated into a microchannel, through experimental and computational analyses. We first demonstrated the ability of the fabricated diagnostic devices to detect DNA-based plant viruses from an infected tomato crop based on the loop-mediated isothermal amplification (LAMP) method. Moreover, we demonstrated the simultaneous detection of RNA-based plant viruses, which can infect cucurbits, by using the reverse transcription LAMP (RT-LAMP) method. The multiplex RT-LAMP assays revealed that multiple RNA viruses extracted from diseased cucumber leaves were successfully detected within 60 min, without any cross-contamination between reaction microchambers, on our diagnostic device.

## 1. Introduction

One of the most essential and urgent technological challenges in agriculture is minimizing crop losses caused by plant diseases and insect pests [1]. For example, the tomato yellow leaf curl virus (TYLCV) is responsible for causing one of the most devastating viral diseases affecting tomato crops in open fields and greenhouses worldwide, resulting in severe yield losses of up to 100% [2]. Cucurbit chlorotic yellows virus (CCYV) transmitted by *Bemisia tabaci* is also a major viral disease that severely affects cucurbit crops (e.g., cucumber, melon, squash, and watermelon). CCYV causes chlorotic leaf spots and the yellowing of leaves, thus not only impairing the quality of fruits and vegetables, but also reducing crop yields by 10% to 20% in a plastic greenhouse [3,4]. As an emerging plant virus, CCYV is currently spreading more widely in many Asian and African countries [5]. Therefore, early detection and accurate diagnosis is a crucial step in controlling plant diseases.

The polymerase chain reaction (PCR) is the most widely used method to amplify specific nucleic acid (DNA/RNA) targets, particularly in biological sciences and clinical medicine, owing to its advantages, such as its high sensitivity, accuracy, and reliability [6]. Real-time PCR, also referred to as quantitative PCR (qPCR), is the most sensitive and reliable method for enabling the detection and precise quantification of a minute amount of target DNA sequence in real time over a wide dynamic range [6]. Moreover, multiplex PCR allows the amplification of different target DNA sequences simultaneously in a single tube. However, it requires careful optimization of the PCR reaction for each target sequence, in order to avoid incorrect amplification and primer dimerization [7]. Additionally, the recent advent of digital PCR (dPCR) has provided a high precision and absolute quantification of nucleic acid target DNA sequences without comparison to standards [8]. Therefore, PCR has also become an indispensable tool for the detection and identification of plant viruses [9,10,11].

As another nucleic acid sequence-based amplification method, loop-mediated isothermal amplification (LAMP) has been developed [12,13]. In the LAMP method, a few copies of the target DNA can be amplified approximately 10^9^ times within 30–60 min by using a set of four to six specially designed LAMP primers. These include two inner primers, FIP and BIP; two outer primers, F3 and B3; and in some cases, one or two loop primers, LF and LB [12]. In general, this technique has been shown to be more sensitive than conventional PCR, but less sensitive than qPCR [14]. Meanwhile, the amplification of nucleic acids can be performed under isothermal conditions (60–65 °C), which only requires a simple hot-water bath instead of expensive instrumentation (i.e., a thermocycler) for precise temperature control and rapid thermal cycling in PCR. Therefore, the LAMP method has considerable potential for providing a cost-effective and easy-to-use diagnostic tool and enabling on-site diagnoses with little equipment [15]. Considering this, we decided that it was more appropriate for LAMP to be integrated into our microfluidic diagnostic device than PCR, in order to enable on-site diagnostic testing without technical expertise.

To date, many attempts have been made to utilize the LAMP method for the diagnosis of plant viral diseases, for example, tomato yellow leaf curl virus (TYLCV) [2], tomato chlorosis virus (ToCV) [16], turnip mosaic virus (TuMV) [17], watermelon mosaic virus (WMV) [18], and squash mosaic virus (SqMV) [18]. However, multiple simultaneous viral infections are quite common in nature, leading to more severe symptoms or even plant mortality in comparison to singly infected plants, owing to their synergistic or antagonistic interactions [19]. Therefore, the early and accurate detection of multiple simultaneous viral infections is a key strategy for realizing the eventual control of plant diseases. In the conventional LAMP assay, it is necessary to prepare and test as many sample/reagent mixtures individually for each targeted plant virus. This requires long and tedious sample preparation and thus, it is unrealistic to expect a person to do it without expert skills and knowledge, as well as on-site diagnostic testing experience. Another problem associated with multiplex diagnosis is the higher operating costs, owing to the consumption of a relatively large amount of LAMP reagents (in general, 10–25 µL for testing a single virus).

Recent progress in microfluidic technology is being harnessed to address such issues. There have been several attempts to perform nucleic acid amplification in microfluidic devices without compromising the specificity and sensitivity. For example, droplet-based microfluidic devices [20,21] enable the highly sensitive quantification of nucleic acids in a sample, but they are inadequate for multiplex diagnosis. In contrast, centrifugal microfluidic compact discs [22,23,24] and stationary chamber-based microfluidic devices [25,26] have the potential for multiplex diagnosis and genotyping pathogens such as bacteria and viruses, but require laborious and multiple operations and/or complex and expensive instrumentation. In this paper, we outline the design and fabrication of a microfluidic diagnostic device capable of autonomous sample mixing and dispensing into an array of reaction microchambers for the simultaneous genetic detection of multiple plant viruses, based on the LAMP method. Moreover, we demonstrate the possibility of the detection of a DNA-based plant virus infecting tomato and simultaneous detection of RNA-based plant viruses infecting cucurbits using fabricated microfluidic devices. This technology offers unique advantages, such as rapid detection, portability, simplicity, and the low-cost diagnosis of a wide variety of plant viruses.

## 2. Materials and Methods

### 2.1. Design of the Multiplex Genetic Diagnostic Device

**Figure 1:**
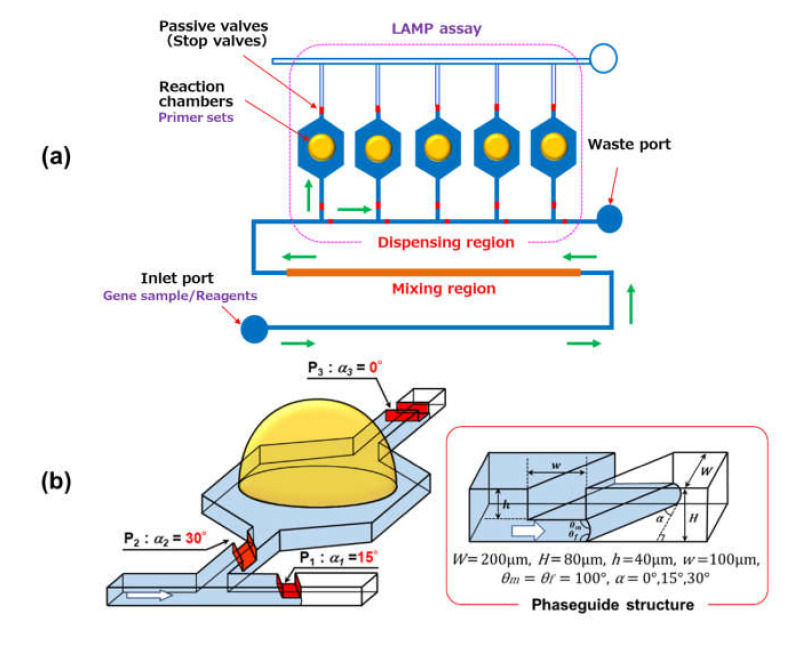

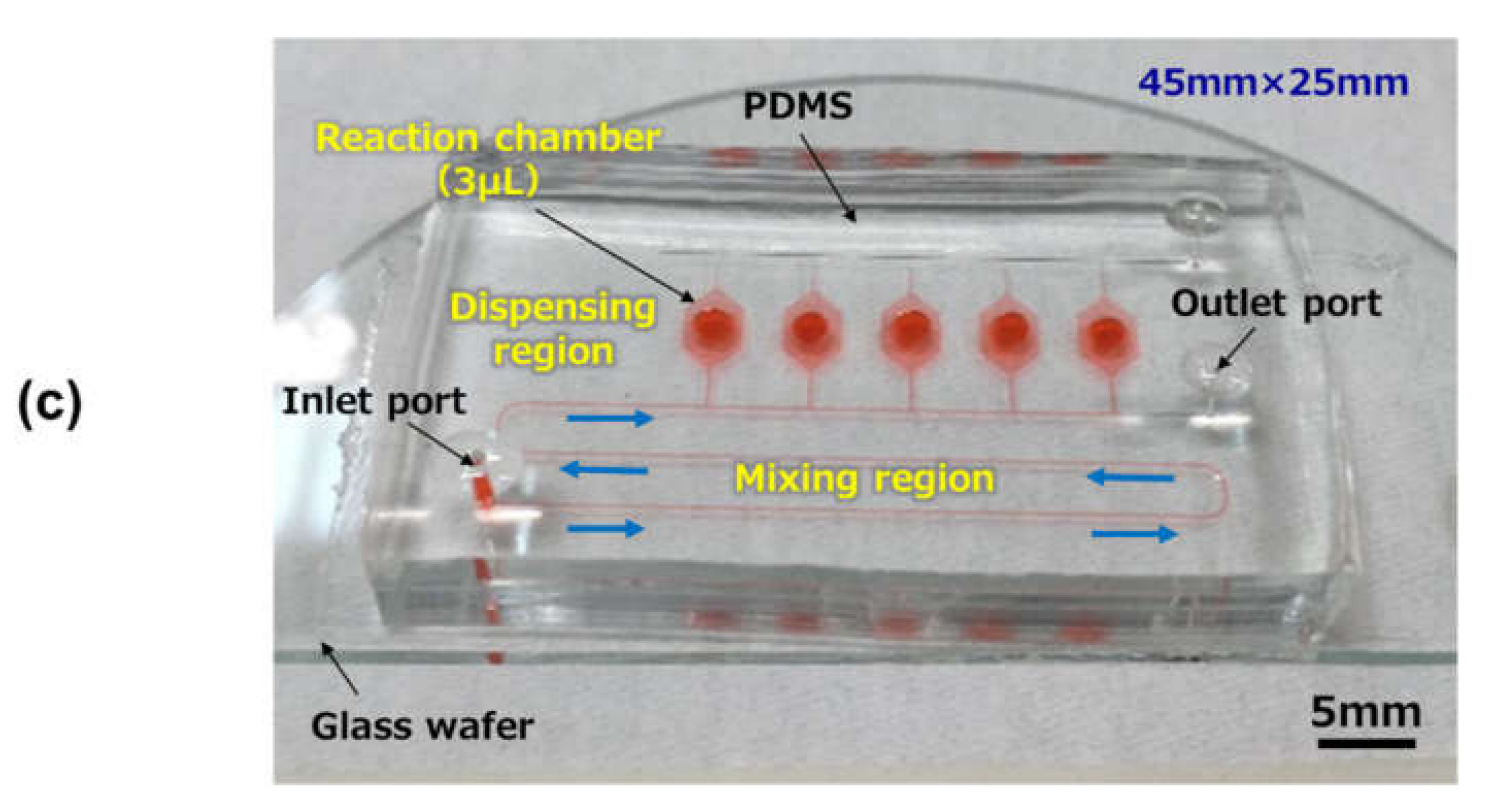
(**a**) Schematic diagram of a parallelized microfluidic device integrated with a chaotic mixer and phaseguide-assisted sample dispensers for multiplex genetic diagnosis. (**b**) Detailed design of the reaction microchamber integrated with three types of phaseguide structures with different inclined angles to be used as passive stop valves to dispense the same volume of sample solution into each microchamber. (**c**) Photograph of the fabricated polydimethylsiloxane (PDMS) microfluidic device filled with red ink.

Figure 1 shows a schematic diagram of the polydimethylsiloxane (PDMS)-based microfluidic device employed for the multiplex genetic diagnosis of plant diseases and pests. The device consists of two main parts: a mixing region and a dispensing region (also used as reaction and detection regions). In this study, nucleic acid samples (DNA or RNA) extracted from plants and/or insects and gene amplification reagents were introduced into a microfluidic channel and efficiently mixed while flowing through the mixing region, in which a simple passive mixer (referred to as a chaotic mixer [27]) was integrated into the microchannel. Next, the sample mixture was precisely dispensed into five reaction chambers located in the dispensing region, in which a set of three stop valves with different burst pressures (referred to as phaseguides [28,29]) were integrated into each microchamber to enable autonomous sample dispensing (see Figure 1b). After filling all of the microchambers, multiple DNA or RNA targets were simultaneously amplified using different primer sets for each targeted nucleic acid, which had been spotted in the reaction chambers beforehand. In our approach, the loop-mediated isothermal amplification (LAMP) method is used for gene amplification owing to its advantages, such as its high specificity and efficiency under isothermal conditions, enabling us to formulate a very simple system design and operation, as described in the introduction [11]. An example of a fabricated PDMS microfluidic device is shown in Figure 1c. The width and height of the microchannel were approximately 200 and 80 µm, respectively. The fabricated microfluidic device was approximately 45 mm × 25 mm in size.

### 2.2. Fabrication Process of the Microfluidic Diagnostic Device

**Figure 2:**
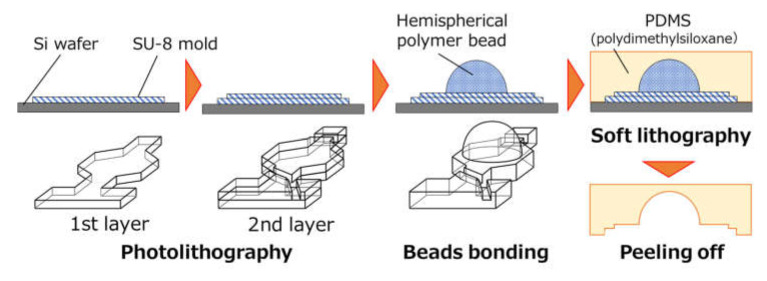
Fabrication process for a PDMS-based microfluidic diagnostic device fabricated by a modified soft-lithography process assisted with hemispherical polymer beads to create deep localized microchamber structures.

The PDMS-based microfluidic devices were fabricated through a modified soft lithography process, using a negative thick photoresist (SU-8 3050, MicroChem Corp., Newton, MA, USA) as a mold. It should be noted that, in this process, hemispherical polymer beads were used to create deep localized microchamber structures required to obtain a sufficient signal strength after the LAMP assay. The fabrication process is described in detail in the Supplementary Material (see Section S1). In brief (see Figure 2), single-crystal silicon wafers (e-Prize Co., Yokohama, Japan) were prepared as a starting material after cleaning in a 4:1 mixture of H_2_O_2_ and H_2_SO_4_ at a temperature above its boiling point for 10 min. SU-8 master molds were then fabricated by a two-step photolithography process. Here, the thicknesses of the first and second layers of the resulting SU-8 patterns were adjusted to be approximately 40 µm each to integrate ridge structures (ca. 40 µm in height) inside a microchannel (ca. 80 µm in height), which were used as a chaotic mixer and phaseguides for sample mixing and dispensing, respectively. The photolithography conditions for SU-8 patterning are described in detail in the Supplementary Material (see Table S1). Hemispherical polymer beads (2 mm in diameter, SAYAKOBO, Yokohama, Japan) were then bonded with epoxy adhesive (Araldite, Huntsman Japan, Kobe, Japan) on the surface of the patterned SU-8 mold to create deep localized microchamber structures. Finally, the microfluidic devices were fabricated by replicating the SU-8 mold pattern glued with hemispherical beads onto PDMS (Silpot 184, Dow Corning Toray Co., Ltd., Tokyo, Japan), which was cured at 80 °C for 40 min. Circular holes (1.0 mm in diameter) for the inlet and outlet ports were punched into the PDMS devices by using a biopsy punch piercing tool (Kai Industries Co. Ltd., Gifu, Japan).

### 2.3. Operating Procedure for the Multiplex LAMP Assay in Microfluidic Diagnostic Devices

**Figure 3:**
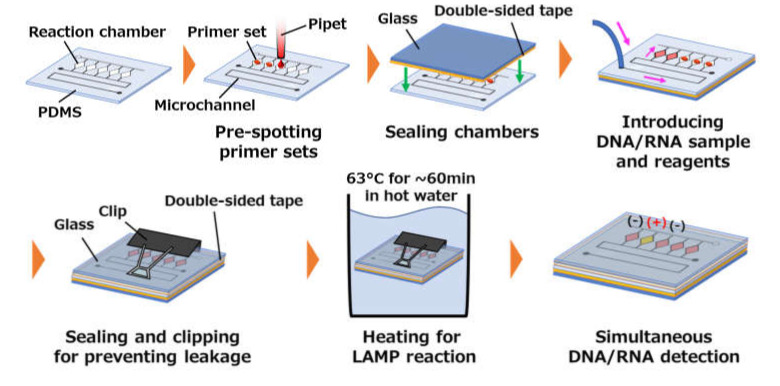
Operating procedure for the multiplex loop-mediated isothermal amplification (LAMP) assay employed in microfluidic diagnostic devices for the simultaneous detection of DNA- and RNA-based plant viruses.

The operating procedure for the multiplex LAMP assay in microfluidic diagnostic devices for the simultaneous detection of DNA- and RNA-based plant viruses was as follows (see Figure 3). First, various specific primer sets (0.5 µL each), expected to amplify viral DNA or RNA attributable to targeted infectious diseases, were pre-spotted and dried in each reaction chamber. Following this, both the microchambers and microchannel were sealed with a glass wafer (AS-3, Toshin Riko Co., Ltd., Tokyo, Japan) via a silicone-based adhesive transfer tape (91022, 3M, St. Paul, MN, USA). After dispensing the mixture of a sample (viral DNA or RNA) and LAMP reagents into five reaction chambers at a flow rate of 5 µL/min with a syringe pump (YSP-201, YMC Co., Ltd., Kyoto, Japan), the inlet and outlet ports were sealed with a glass wafer via silicone-based adhesive double-sided tape (No. 5303W, Nitto Denko Corp., Osaka, Japan). The microfluidic device was further clipped mechanically to prevent leakage at the glass/PDMS/glass interface and then immersed in a hot-water bath (TB-1NC, AS ONE, Osaka, Japan) to amplify the targeted DNA or RNA through the LAMP or reverse transcription LAMP (RT-LAMP) reaction, respectively, under isothermal conditions at 63 °C for 20–60 min.

The LAMP reagents consisted of a mixture of 20 mM Tris-HCl (pH 8.0), 10 mM KCl, 10 mM (NH_4_)_2_SO_4_, 8 mM MgSO_4_, 0.1% Tween 20 (all chemicals were purchased from FUJIFILM Wako Pure Chemical Corp., Osaka, Japan), 0.8 M betaine (Sigma-Aldrich, Darmstadt, Germany), and 1.0 mM of each dNTP (GE Healthcare, Chicago, IL, USA) containing 8 U of *Bst* DNA polymerase (Nippon Gene Co., Ltd., Tokyo, Japan). For the detection of RNA-based viruses, 1 U of AMV reverse transcriptase (Promega, Madison, USA) was added to the LAMP reagents. All of the primer sets used in the LAMP assay, one for a DNA-based virus for tomato and four for RNA-based viruses for cucurbits (as described in Section 3.4), were designed and provided by Aichi Agricultural Research Center (Aichi, Japan). A primer mixture for each virus included 1.6 µM of each inner primer (FIP and BIP); 0.2 µM of each outer primer (F3 and B3); and, in some cases, a further addition of 0.8 μM of each of the one or two loop primers (LF and LB). The total RNA (containing the viral RNA target) extracted from infected cucumber leaves was assessed using a Qubit 4 Fluorometer (Thermo Fisher Scientific Inc., Waltham, MA, USA).

In the experiments, berberine chloride hydrate (FUJIFILM Wako Pure Chemical Corp., Osaka, Japan) was chosen as the fluorescence indicator of DNA/RNA amplification in the LAMP reactions as it is a non-toxic natural dye derived from plants [30]. The final concentration of berberine was adjusted to 180 µM in a mixture of a sample and LAMP reagents. It should be noted that berberine is a DNA-intercalating dye which increases in green fluorescence after binding to DNA and has an excitation/emission wavelength of around 450/530 nm, respectively [30,31]. Fluorescence imaging was performed using an inverted microscope (TE2000-U, Nikon, Tokyo, Japan) equipped with a charged coupled device (CCD) camera (Digital Sight DS-Fi2, Nikon, Tokyo, Japan) and an objective lens (CFI Plan Apochromat Lambda 2×, Nikon, Tokyo, Japan) under blue-violet light irradiation in the 400–440 nm range, which was selected with an optical filter (BV-2A, Nikon, Tokyo, Japan) from a mercury light source (C-SHG1 Super High Pressure Mercury Lamp Power Supply, Nikon, Tokyo, Japan).

## 3. Results and Discussion

### 3.1. Fabrication of the Microfluidic Diagnostic Device

**Figure 4:**
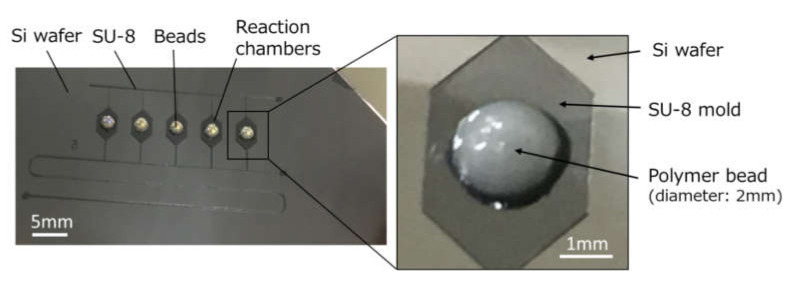
Photographs of an SU-8 master mold pattern on an Si wafer, in which hemispherical polymer beads (2 mm in diameter) were bonded with epoxy adhesive on the surface of five microchamber patterns to create deep localized structures, after replicating this mold in PDMS.

**Figure 5:**
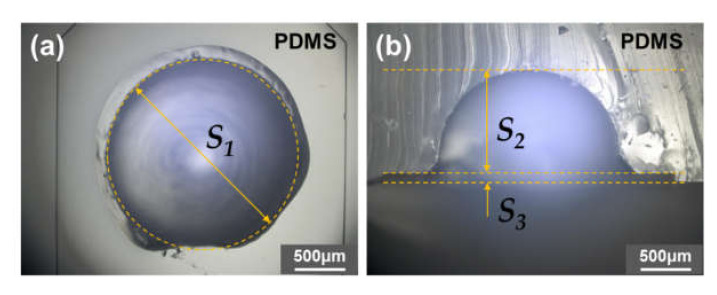
Optical microscopy images showing (**a**) close-up plane and (**b**) cross-sectional views of a PDMS reaction chamber precisely replicated from a hemispherical polymer bead glued on the SU-8 pattern.

Figure 4 shows photographs of an SU-8 master mold, consisting of an array of five microchambers and a microchannel forming a network connecting them, patterned on an Si wafer by a two-step photolithography process. Additionally, hemispherical polymer beads were glued in the center on the top surface of each SU-8 chamber pattern. Figure 5 shows close-up plane and cross-sectional views of a PDMS reaction chamber, replicated from a hemispherical polymer bead glued on the SU-8 pattern, taken with a digital microscope (VHX-2000, Keyence, Osaka, Japan). The dimensions of the replicated PDMS structure denoted by symbols *S*_1_, *S*_2_, and *S*_3_ were estimated to be 1924 ± 41 µm, 1091 ± 46 µm, and 78.5 ± 6.5 µm (n = 40), respectively, which represent the mean ± standard deviation of 40 experimental measurements. Here, *S*_1_ and *S*_2_ were determined by the diameter and height of the hemispherical polymer bead, respectively, and *S*_3_ was determined by the height of the SU-8 chamber patterns (i.e., the total height of the first and second SU-8 layers). It should also be noted that the first layer of SU-8 patterns was *S*_4_ = 37.4 ± 3.3 µm (n = 40) in height, which determines the height of the ridge structures (a chaotic mixer and phaseguide-based stop valves) embedded in a microchannel, that is, *S*_3_ − *S*_4_ is equivalent to the height of the ridge structures.

**Figure 6:**
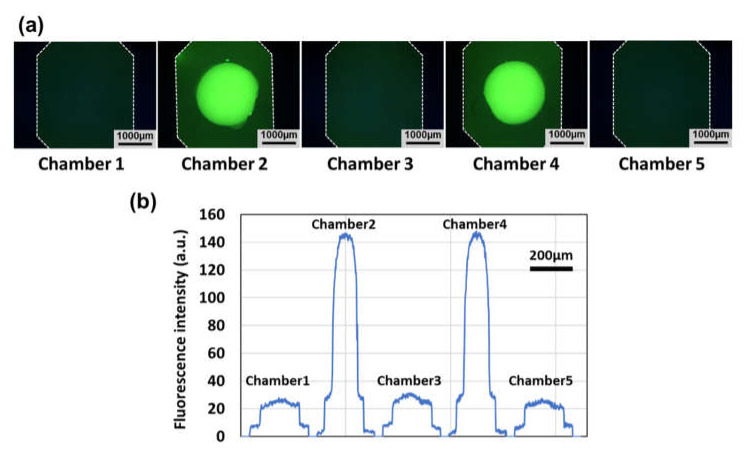
A comparison of the fluorescence intensity of two kinds of microchambers replicated with or without a hemispherical polymer bead. (**a**) Fluorescent microscopy images of microchambers (gain: 1×, exposure time: 2 s). (**b**) Cross-sectional profiles of the fluorescence intensity across the center of each microchamber.

A preliminary experiment was conducted to compare the fluorescence intensity of two types of microchambers replicated with or without a hemispherical polymer bead, as shown in Figure 6. In the experiment, fluorescein (excitation/emission wavelength: 494/521 nm, Tokyo Chemical Industry Co., Ltd., Tokyo, Japan), which was used as a fluorescent indicator, was adjusted to a concentration of 1 mM in 25 % dimethyl sulfoxide (DMSO, Sigma-Aldrich, Darmstadt, Germany), and then dispensed in each microchamber. Fluorescence measurements were conducted using an inverted microscope (TE2000-U, Nikon) under blue light irradiation (450–490 nm in wavelength) selected from a mercury light source (C-SHG1, Nikon) with an optical filter (B-2A, Nikon). As shown in Figure 6a, the microchambers (Nos. 1, 3, and 5) replicated without a bead exhibited almost no detectable fluorescence increase compared to that observed outside the microchamber area, probably owing to its shallow depth (ca. 80 µm in depth). In contrast, very bright green fluorescence was observed in the microchambers (Nos. 2 and 4) replicated with a bead. The cross-sectional profiles of the fluorescence intensity quantified using the ImageJ software (version 1.52a, National Institutes of Health, USA) revealed that the fluorescence intensity increased more than four-fold owing to an increase in the depth of the microchamber of almost fifteen-fold (see Figure 6b). Although the volume of each microchamber increased from 0.9 to 3.1 µL, this sample/reagent volume is still small compared to that used in a standard LAMP protocol (25 µL), allowing us to reduce the consumption of sample/reagent.

### 3.2. Evaluation of the Mixing Efficiency for Different Types of Chaotic Mixers

The simple and effective mixing of a sample containing targeted nucleic acids (DNA or RNA) and reagents for the LAMP assay is a particularly important process that directly affects the amplification efficiency and sensitivity of detection of the DNA or RNA targets. In our experiments, a sample pre-mixed with reagents was introduced into the fabricated microfluidic devices through a single inlet port (described below) to evaluate the possibility of enabling autonomous sample dispensing into an array of microchambers and the amplification of targeted nucleic acids in our diagnostic devices. However, one of our goals was to realize a fully automated microfluidic diagnostic system, in which reagents stored in a reservoir are automatically introduced independently of the sample containing viral DNA or RNA. Another goal was to enable point-of-care (POC) diagnosis, in which reagents would be pre-fixed inside a microchannel to provide a simpler microfluidic system. Such a strategy would allow us to ensure the rapid onsite detection of nucleic acid targets, wherein the operator is only needed to introduce a sample into the diagnostic device. Therefore, in any case, an effective mixer needed to be integrated into a microchannel.

It is well-known that fluid flow is dominated by laminar flow in microchannels owing to their very low Reynolds numbers, thus making it difficult to mix fluids. Some mechanically moving active mixers in microchannels and external forces, such as electrical, magnetic, and sound fields, allow effective mixing, but the systems and equipment involved are complicated and costly [32]. Among the different types of micromixers, a chaotic mixer (categorized as a passive micromixer) is the most appropriate for our proposed device owing to its simple geometrical configuration and high mixing efficiency, in which periodic ridge structures can generate a transverse flow in a microchannel [27]. Therefore, we next estimated the mixing efficiency of chaotic mixers with different types of periodic ridge structures embedded in a microchannel through experimental and computational analyses.

#### 3.2.1. Experimental Investigation of Mixing Phenomena in a Microchannel

**Figure 7:**
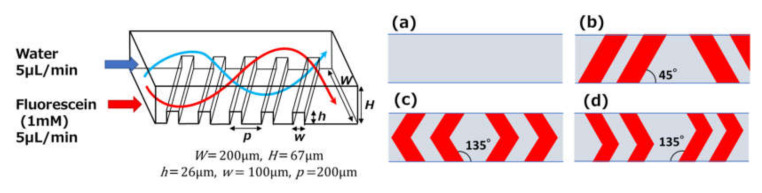
Schematic diagram of chaotic mixing (illustrated by the streamlines of two fluids) in a microchannel and designs of three types of chaotic mixers. The mixing efficiency was estimated in (**a**) a rectangular microchannel without ridges, which was used for comparison, and (**b**) a chaotic mixer with periodic oblique, (**c**) symmetric V-shaped, and (**d**) asymmetric V-shaped ridges embedded in the microchannel.

For these experiments, microfluidic devices with two inlet ports were prepared to evaluate the mixing efficiency of three different types of chaotic mixers, as well as a rectangular microchannel without ridges, which was investigated for comparison. The geometrical configurations and dimensions of the chaotic mixers are shown in Figure 7. Each type of chaotic mixer consisted of four sets of 35 periodic ridge structures (100 µm in width, 26 µm in height, and 200 µm in pitch), which were fabricated inside a PDMS microchannel (200 µm in width and 67 µm in height); that is, the total number of ridges (140) was equivalent to approximately a 30 mm channel length. Additionally, the angle of each set of ridge structures was reversed with respect to the flow direction in the microchannel.

**Figure 8:**
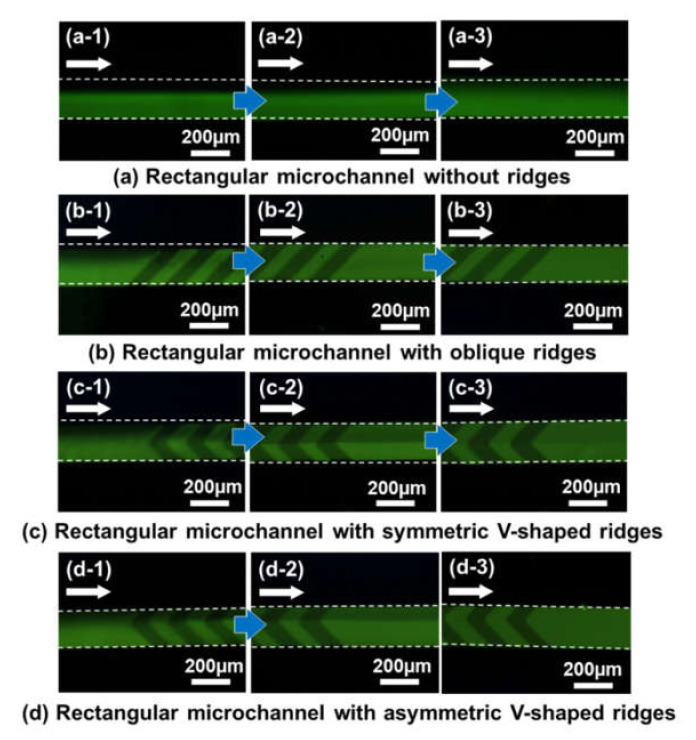
Experimental results showing fluorescence microscopy images (taken with an optical filter B-2A, gain: 1×, exposure time: 2 s) of the mixing process in (**a**) a rectangular microchannel without ridges, and (**b**) a chaotic mixer with oblique ridges, (**c**) symmetric V-shaped ridges, and (**d**) asymmetric V-shaped ridges. Fluorescein-dyed water (1 mM) and pure water were introduced at the same flow rate of 5 µL/min.

Figure 8a shows fluorescence microscopy images of the flow behavior of two different fluids in a rectangular microchannel without any ridges, in which fluorescein-dyed water (1 mM) and pure water were introduced into the microchannel from individual inlet ports at the same flow rate of 5 µL/min. As expected, a laminar flow was observed in the microchannel without a chaotic mixer owing to its low Reynolds number. Figure 8b shows the mixing behavior in a microchannel embedded with periodic oblique ridges before passing through the first set of ridges (b-1), after passing through the first one (b-2), and after passing through the fourth one (b-3). Green fluorescence seems to be of a uniform intensity across the microchannel width, even after passing through the first one, equivalent to crossing approximately 7 mm in channel length. The mixing behaviors in microchannels with symmetric and asymmetric V-shaped ridges are shown in Figure 8c and Figure 8d, respectively. In both types of chaotic mixers, the interface between the two fluids was still observed above and below the apex of the V-shaped structures only after passing through the first set of ridges.

To quantify the mixing efficiency in the various chaotic mixers described above, the standard deviation of fluorescence intensities over the microchannel cross-section was estimated using the ImageJ software, and the mixing efficiency *η* (%) was then calculated using the following Equation (1):

(1) $$\eta \;\left(\%\right)=\left(1-\frac{SD_{x}}{SD_{0}}\right)\times 100,$$

where the symbols *SD*_0_ and *SD_x_* are the standard deviations estimated at a position just before passing through the first set of periodic ridges (defined as the initial position) and at an arbitrary position *x* (mm) (defined as the mixing distance from the initial position) along the flow direction in a microchannel, respectively.

**Figure 9:**
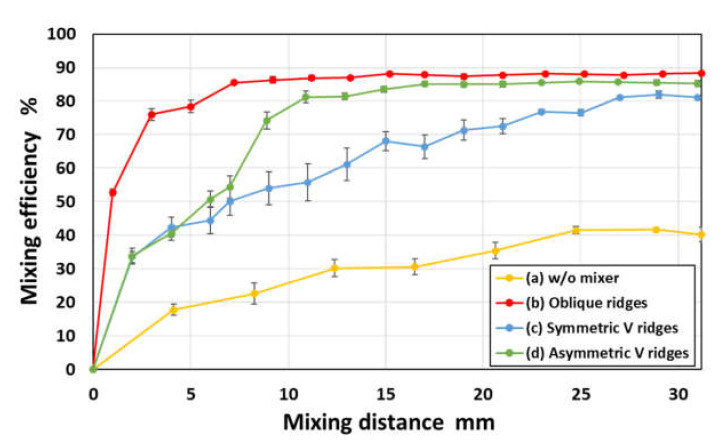
Experimental results showing the mixing efficiency of three different types of chaotic mixers with periodic oblique, symmetric V-shaped, and asymmetric V-shaped ridges as a function of the mixing distance along the flow direction. For comparison, the data obtained in a rectangular microchannel without ridges is also plotted in the graph. Note that each point and error bar in the plot represent the mean values and their standard deviations, respectively.

In Figure 9, the resulting mixing efficiencies are plotted as a function of the mixing distance along the flow direction, in which the vertical bars represent the standard deviation for each mixer in the three experiments. The mixing efficiency in a rectangular microchannel without any mixers reached only 40% after the two fluids had passed through the microchannel at a mixing distance of 30 mm. In contrast, all of the chaotic mixers showed an acceptable mixing performance (mixing efficiency of 80% or above) within a mixing distance of 30 mm. Among them, the chaotic mixer with simple oblique ridge structures exhibited the highest mixing efficiency of almost 90% within a mixing distance of 10 mm. Therefore, this type of chaotic mixer was determined to have an optimum design and was integrated into the mixing region of the microfluidic diagnostic devices developed here.

#### 3.2.2. Computational Analysis of Mixing Phenomena in a Microchannel

**Figure 10:**
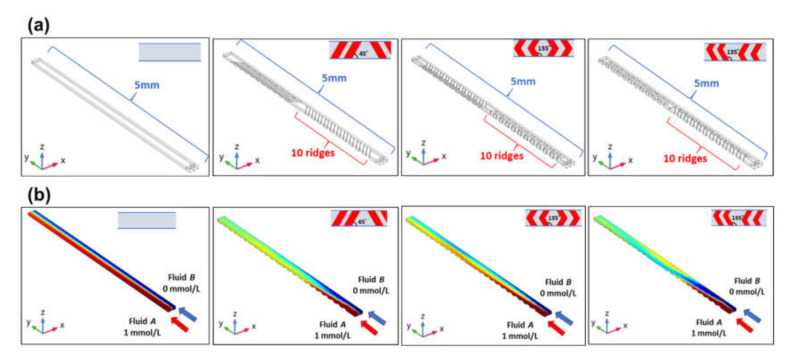
(**a**) Three-dimensional FEM models showing a rectangular microchannel without any ridge structures, and with periodic oblique ridges, symmetric V-shaped ridges, and asymmetric V-shaped ridges. (**b**) Simulation results showing the concentration contours of two different fluids (with a concentration of 0 and 1 mmol/L) in each microchannel at a flow velocity of 0.0128 m/s each (equivalent to 5 µL/min).

To further understand the mixing behavior in chaotic mixers, a computational analysis was performed using the finite element method (FEM) on commercially available software (COMSOL Multiphysics version 5.4, COMSOL AB, Stockholm, Sweden). To reduce the computational resources and time, a three-dimensional FEM model (approximately 80,000 elements) for each chaotic mixer was simplified by reducing the number of periodic ridge structures and their sets, as shown in Figure 10a, in which each chaotic mixer consists of two sets of 10 periodic ridge structures (100 µm in width, 26 µm in height, and 200 µm in pitch) in a rectangular microchannel (200 µm in width, 67 µm in height, and 5 mm in length). It should be noted that the geometrical dimensions of the ridge structures and microchannel cross-section were similar to the dimensions of the microfluidic devices used in the mixing experiments above.

The chaotic mixing phenomena were analyzed under steady-state conditions by coupling the single-phase flow and transport of the diluted species modules of COMSOL. The fluid was assumed to be incompressible, and a no-slip boundary condition was imposed on the surface of the microchannel wall. Figure 10b shows the simulation results highlighting the concentration contours of two different fluids in a rectangular microchannel without any mixer or with three different types of chaotic mixers. To consider the mixing of two fluids, red-colored fluid *A* (with a concentration of 1 mmol/L) and blue-colored fluid *B* (with a concentration of 0 mmol/L) were introduced into a microchannel at a flow velocity of 0.0128 m/s each, which is equivalent to the 5 µL/min used in the experiments above. The pressure at the outflow boundary was set to zero. A diffusion coefficient of 4.25 × 10^–10^ m^2^/s at 25 °C for fluorescein [33] was used in the numerical simulations. Assuming the fluids to be water, the density and dynamic viscosity of the two fluids were set to 1.0 × 10^3^ kg/m^3^ and 1.0 × 10^–3^ kg/m·s, respectively. As shown in Figure 10b, laminar flow occurred in a microchannel without a chaotic mixer because of a low Reynolds number. In contrast, three-dimensional twisting flow occurred in a microchannel with a chaotic mixer.

**Figure 11:**
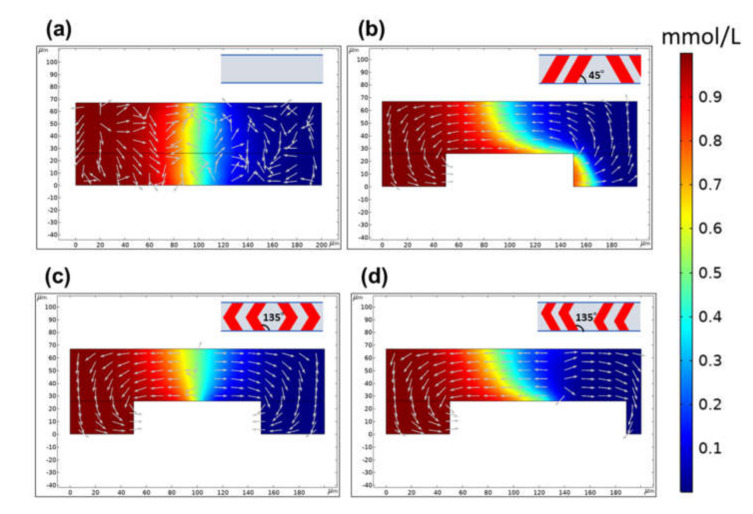
The concentration distribution (contour) and transverse velocity fields (arrows) across the microchannel cross-section at the position *y* = 0.45 mm away from the inlet. (**a**) Rectangular microchannel without ridges. (**b**) Chaotic mixer with oblique ridges, (**c**) symmetric V-shaped ridges, and (**d**) asymmetric V-shaped ridges.

Figure 11a shows the concentration distribution (contour) and transverse velocity fields (arrows) across the cross-section of a rectangular microchannel without a mixer at the position *y* = 0.45 mm away from the inlet. The concentration contour demonstrated that the interface between the two fluids was clearly visible due to laminar flow. Additionally, two symmetric vortices appeared on the left and right sides of the cross-section of a microchannel according to the transverse velocity profiles. In contrast, a single streamwise vortex seemed to spread over the entire cross-sectional area of a microchannel with a chaotic mixer displaying periodic oblique ridges. The progress of mixing could be observed, as shown in Figure 11b. Figure 11c,d shows the mixing behavior of a chaotic mixer with symmetric and asymmetric V-shaped ridges, respectively. Like the microchannel without a mixer (see Figure 11a), two opposite vortices were generated on the left and right sides of the cross-section of a microchannel, in which their boundaries coincided with the position of the apex of the V-shaped ridge structures.

**Figure 12:**
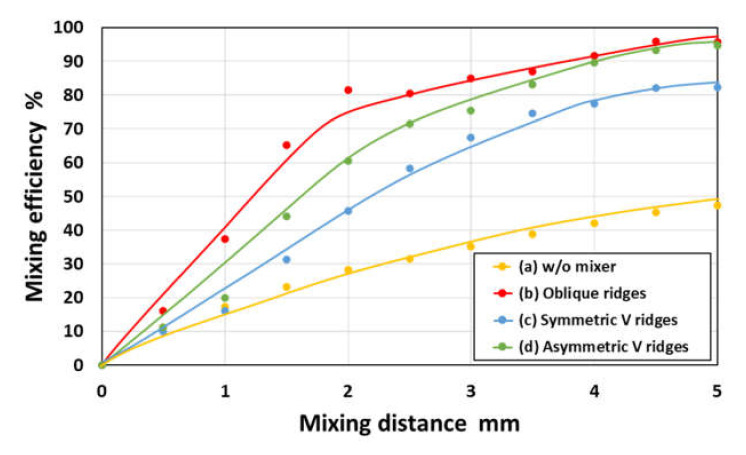
Simulation results showing the mixing efficiency of three different chaotic mixers with periodic oblique, symmetric V-shaped, and asymmetric V-shaped ridges as a function of the mixing distance along the flow direction. For comparison, the data obtained in a rectangular microchannel without ridges is also plotted in the graph.

The mixing efficiencies of each chaotic mixer estimated from the simulations are plotted in Figure 12. Although the mixing efficiencies increased within much shorter mixing distances compared to those of the experimental results (see Figure 9), the simulation results can qualitatively describe the differences in mixing efficiencies between the three different types of mixers. It should be noted that the slight decrease in the mixing efficiency in the V-shaped ridges compared to the simple oblique ridges is most likely because the fluids were divided into two flow patterns in a microchannel, as mentioned above (see Figure 11c,d). This suggests that a chaotic mixer with highly asymmetric structures against the flow direction could exhibit effective mixing behavior.

### 3.3. Investigation of Autonomous Sample Dispensing with Phaseguides

To enable multiplex diagnosis in microfluidic devices, a mixture of a sample (viral DNA or RNA) and reagents for the LAMP assay should be precisely dispensed into multiple reaction chambers. We proposed a simple and autonomous dispensing system without the use of movable mechanical parts and external energy. Figure 1b shows the design of a reaction chamber integrated with a set of three different phaseguide structures with varying angles of inclination against the flow direction, which can dispense the same volume of fluids sequentially into each reaction chamber. It is well-known that phaseguides can act as pressure barriers due to the meniscus pinning effect [28,29], as illustrated in Figure 1b. In theory, the burst pressure can be derived from the Young–Laplace equation, that is, as $\Delta P=\gamma \left(1/R_{2}+1/R_{2}\right)$, where Δ*P* is the pressure difference across the fluid interface, *γ* is the surface tension of water, and *R*_1_ and *R*_2_ are the principal radii of the surface curvature. For a rectangular microchannel with a phaseguide ridge with different inclined angles *α* (deg.), the burst pressure *P_phg_* (Pa) can be expressed as follows:

(2) $$P_{phg}=-\gamma \left(\frac{2\cos\theta_{m}\cos\alpha }{W}+\frac{\cos\theta_{m}+\cos\theta_{f}}{H-h}\right),\;\;\alpha \neq 90{}^{\circ}$$

where *W* and *H* are the width and height of a rectangular microchannel, respectively; *h* is the height of the phaseguide ridge; *θ_m_* is the water contact angle for the sidewall surfaces of the microchannel and the top surface of the phaseguide ridge (i.e., PDMS); and *θ_f_* is the water contact angle for the bottom surface of the microchannel (i.e., silicone-based adhesive tape). The inclined angle *α* is defined as the angle between the phaseguide ridge and the sidewall of the microchannel. For the microfluidic devices fabricated here, the burst pressures in a microchannel (*H* = 80 µm, *W* = 200 µm for phaseguides *P*_1_ and *P*_2_, and *W* = 100 µm for phaseguide *P*_3_) integrated with a phaseguide ridge (*h* = 40 µm) were calculated to be *P*_3_ = 2.37 kPa, *P*_1_ = 2.06 kPa, and *P*_2_ = 1.94 kPa for the different inclined angles of *α* = 0°, 15°, and 30°, respectively, where the surface tension of water was 0.073 N/m [29] and the water contact angles were *θ_m_* = *θ_f_* = 100°.

**Figure 13:**
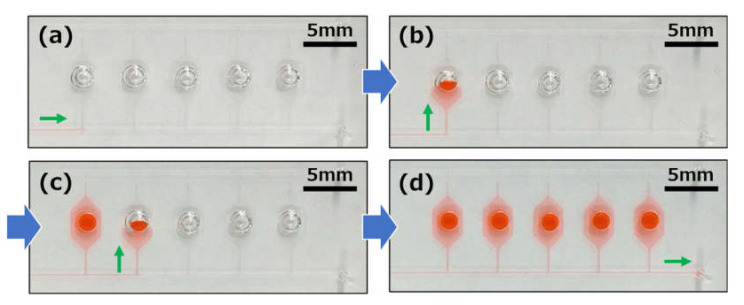
Experimental results showing the performance of autonomous sample dispensing into each reaction chamber using three types of phaseguide structures with different inclined angles against the flow direction. (**a**) The flow of water was stopped after reaching the phaseguide P_1_. (**b**) The flow direction was changed toward the first microchamber by passing through the phaseguide P_2_. (**c**) The flow toward the second microchamber occurred by passing through the phaseguide P_1_. (**d**) All of the microchambers were autonomously filled with water sequentially by repeating this process.

Figure 13 shows a typical experimental result that demonstrates liquid being sequentially dispensed into an array of five microchambers, where water colored with red food coloring (0.1% w/v) was introduced into a microchannel with a syringe pump at a flow rate of 5 μL/min. The dispensing procedure was as follows. First, the flow of water was stopped after reaching the phaseguide *P*_1_ (see Figure 13a), and the flow direction was then changed toward the microchamber by passing through the phaseguide *P*_2_, owing to the burst pressure *P*_2_ < *P*_1_ (see Figure 13b). After the microchamber was filled with water, the flow of water was stopped at the phaseguide *P*_3_, and the flow toward the second microchamber then occurred by passing through the phaseguide *P*_1_, owing to the burst pressure *P*_1_ < *P*_3_ (see Figure 13c). By repeating this process, all of the microchambers could be autonomously filled with water (see Figure 13d). This result reveals that a high-precision liquid operation can be possible by controlling the different burst pressures at three different phaseguide angles (see Video S1 in the Supplementary Material).

### 3.4. LAMP Assay in the Microfluidic Diagnostic Device

#### 3.4.1. Detection of a DNA-Based Plant Virus

**Figure 14:**
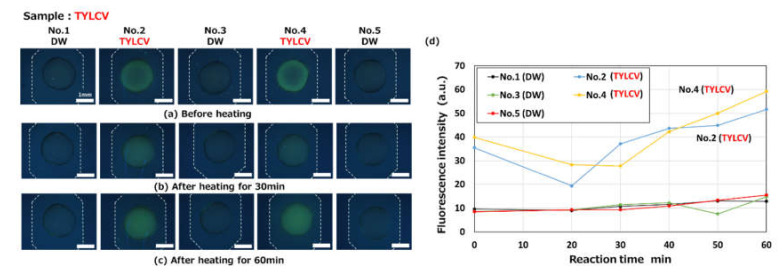
Fluorescence microscopy images showing the LAMP detection of a DNA-based plant virus (tomato yellow leaf curl virus, TYLCV) observed (**a**) before heating and after heating for (**b**) 30 min and (**c**) 60 min at 63 °C (taken with an optical filter BV-2A, gain: 1×, exposure time: 2 s). A primer set for the detection of TYLCV was pre-spotted in reaction chambers (Nos. 2 and 4). (**d**) Changes in fluorescence intensities as a function of the reaction time.

We first demonstrated the use of the LAMP method to detect DNA-based plant viruses, that is, tomato yellow leaf curl virus (TYLCV) Israel strain, which is one of the most devastating plant diseases affecting tomato production worldwide. The infectious clone of TYLCV-Is (pRI-IS) was extracted and purified from *E. coli* cells [34] to be used as a nucleic acid sample. Figure 14a shows fluorescence microscopy images taken before and after the LAMP assay in the fabricated microfluidic diagnostic device. A primer set for the detection of TYLCV-Is (unpublished data; Suzuki et al., 2016) was pre-spotted and dried in the reaction chambers (Nos. 2 and 4), while distilled water (DW) was pre-spotted in the other reaction chambers (Nos. 1, 3, and 5) as a negative control. After a mixture of a sample (containing the viral DNA target) and the LAMP reagents was autonomously dispensed into the five reaction chambers, the LAMP assay was conducted at 63 °C for 20–60 min in a hot-water bath. Before heating, the strong green fluorescence of berberine was already visible in chambers No. 2 and 4. This unfavorable result was most likely caused by the binding of berberine to the single-stranded primers that had been pre-spotted in the chambers [30]. However, the fluorescence gradually decreased, probably due to the fact that the berberine–primer complex, which was generated in the vicinity of the surface of the chamber at an early stage, and evenly diffused throughout the chamber, thus resulting in a decrease in its fluorescence intensity.

As shown in Figure 14d, the fluorescence intensities decreased once at a 20 min reaction time and then markedly increased after 30 min. In contrast, the fluorescence intensities in the other chambers (Nos. 1, 3, and 5 used as a negative control) were low, even after 60 min. It should be noted that there was no evidence of cross-contamination between the chambers. Additionally, the fluorescence of berberine could only be clearly observed in an area replicated from a hemispherical bead. This result suggests that the modified soft-lithography technique proposed here is necessary for the visualization of LAMP assay products in microfluidic devices.

As a preliminary experiment (see Section S2 in the Supplementary Material), we first optimized the detection conditions of target DNA amplified by the LAMP method, that is, an ideal combination of DNA-binding fluorescent dyes (berberine chloride and berberine sulfide) and the appropriate excitation wavelength for each dye (three optical filters: BV-2A, B-2A, and UV-2A for UV irradiation at 330–380 nm). The specifications of the optical filters used are listed in Table S2 in the Supplementary Material. As a result, a combination of berberine chloride and an optical filter BV-2A was determined to be the most appropriate for the LAMP assay in microfluidic diagnostic devices (see Figure S1 and Table S3 in the Supplementary Material). Furthermore, to simplify the fluorescence imaging in the LAMP assays, we demonstrated that the fluorescence microscope can be replaced by a smartphone modified with homemade equipment (see Figures S2 and S3 in the Supplementary Material). The detailed experimental methods are described in the Supplementary Material (see Section S3).

#### 3.4.2. Simultaneous Detection of RNA-Based Plant Viruses

Finally, we explored the possibility of the simultaneous detection of RNA-based plant viruses, which can infect cucurbits, on microfluidic devices. Four types of primer sets were prepared for these experiments, that is, melon yellow spot virus (MYSV) [35], cucurbit chlorotic yellows virus (CCYV) [unpublished data; Fukuta et al., 2009], kyuri green mottle mosaic virus (KGMMV) [36], and cucumber mosaic virus (CMV) [37]. As a sample, total RNAs were extracted from cucumber leaves infected with MYSV or CCYV, which are destructive cucurbit viruses that cause considerable losses in melon and cucumber fields [38,39].

**Figure 15:**
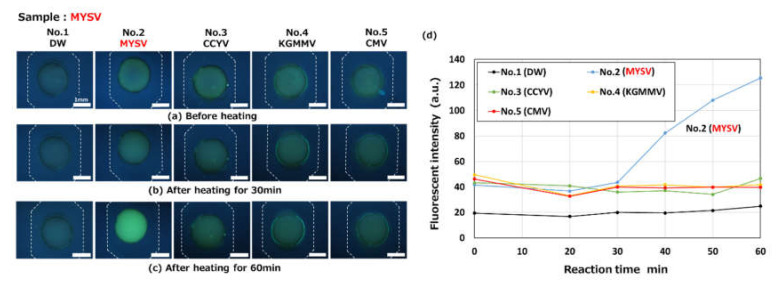
Fluorescence microscopy images showing the reverse transcription LAMP (RT-LAMP) detection of an RNA-based plant virus (melon yellow spot virus, MYSV) observed (**a**) before heating and after heating for (**b**) 30 min and (**c**) 60 min at 63 °C (taken with an optical filter BV-2A, gain: 1×, exposure time: 2 s). A primer set for the detection of MYSV was pre-spotted in a reaction chamber (No. 2). (**d**) Changes in fluorescence intensities as a function of the reaction time. The concentration of total RNA, containing the MYSV viral RNA target, was 22.2 ng/µL after mixing with the LAMP reagents.

Figure 15 shows the results for the detection of MYSV (viral RNA) by the RT-LAMP method on the fabricated microfluidic device. Total RNA containing the MYSV viral RNA target was mixed with LAMP reagents to a concentration of 22.2 ng/µL and introduced into the device. Four different primer sets for MYSY (target for detection), CCYV, KGMMV, and CMV were pre-spotted in the reaction chambers denoted by No. 2 (target for detection), 3, 4, and 5, respectively, while DW was pre-spotted in reaction chamber 1 as a negative control. The LAMP assay was conducted at 63 °C for 20–60 min in a hot-water bath. As shown in Figure 15a, the green fluorescence of berberine appeared in all of the chambers except for chamber 1 (negative control), even before heating. There were likely no primers to bind to berberine in chamber 1, as mentioned previously. As expected, the fluorescence intensity only markedly increased in chamber 2 (MYSV) after heating for more than 40 min (see Figure 15d).

**Figure 16:**
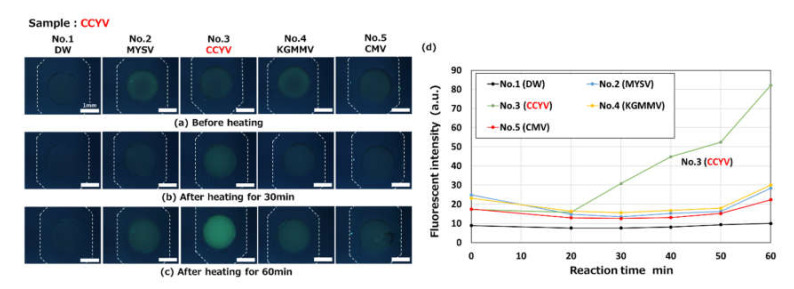
Fluorescence microscopy images showing the RT-LAMP detection of an RNA-based plant virus (cucurbit chlorotic yellows virus, CCYV) observed (**a**) before heating and after heating for (**b**) 30 min and (**c**) 60 min at 63 °C (taken with an optical filter BV-2A, gain: 1×, exposure time: 2 s). A primer set for the detection of CCYV was pre-spotted in a reaction chamber (No. 3). (**d**) Changes in fluorescence intensities as a function of the reaction time. The concentration of total RNA, containing the CCYV viral RNA target, was 19.3 ng/µL after mixing with the LAMP reagents.

Figure 16 shows the results for the detection of CCYV (viral RNA), where four different kinds of primer sets and DW (negative control) were pre-spotted in each reaction chamber in the same way as in the experiment shown in Figure 15. The concentration of total RNA used, containing the CCYV viral RNA target, was 19.3 ng/µL after mixing with LAMP reagents. This was introduced into a microfluidic device. After heating for more than 30 min, the amplification of only the targeted viral RNA, that is, CCYV in chamber 3, could be clearly visualized by an increase in the green fluorescence of berberine.

Additionally, the results of LAMP assays for CMV (viral RNA) and KGMMV (viral RNA), which were extracted from infected celery leaves and cucumber leaves, respectively, are shown in the Supplementary Material (see Section S4). As expected, when the total RNA (17.6 ng/µL), containing the CMV viral RNA target, was introduced into a microfluidic device, the fluorescence intensity only markedly increased in chamber 5 (CMV) after heating for more than 20 min (see Figure S4 in the Supplementary Material). However, in the experiment with 19.0 ng/µL total RNA containing the KGMMV viral RNA target, it showed false positive amplification in chamber 3 (CCYV), as well as a true positive in chamber 4 (KGMMV) (see Figure S5a in the Supplementary Material). However, when the concentration of total RNA was decreased to 3.0 ng/µL, there was no false positive amplification (see Figure S5b in the Supplementary Material). At this lower concentration, the fluorescence intensity only markedly increased in chamber 4 (KGMMV) after heating for more than 30 min. These results indicate that optimization of the concentration of viral RNA target in a sample is particularly important for ensuring that the LAMP reaction is successfully performed. Therefore, in future work, we will further investigate the effect of the concentration of viral RNA on the LAMP reactions.

**Figure 17:**
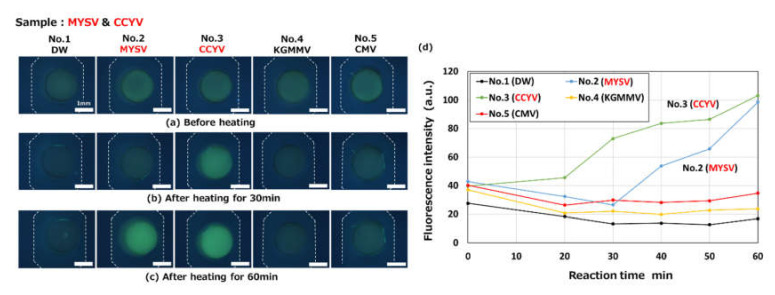
Fluorescence microscopy images showing the simultaneous RT-LAMP detection of multiple RNA-based plant viruses (MYSV and CCYV) observed (**a**) before heating and after heating for (**b**) 30 min and (**c**) 60 min at 63 °C (taken with an optical filter BV-2A, gain: 1×, exposure time: 2 s). Primer sets for the detection of MYSV and CCYV were pre-spotted in reaction chamber Nos. 2 and 3, respectively. (**d**) Changes in fluorescence intensities as a function of the reaction time. The concentrations of total RNA, containing the MYSV and CCYV viral RNA targets, were 11.1 and 9.6 ng/µL, respectively, after mixing with the LAMP reagents.

Figure 17 shows the results of the simultaneous detection of multiple viral RNAs (MYSY and CCYV), where a mixture containing both the viral RNAs as a sample and the LAMP reagents was introduced into a microchannel and dispensed into each reaction chamber. The concentrations of total RNA containing the MYSY and CCYV viral RNA targets in the mixture were 11.1 and 9.6 ng/µL, respectively. The fluorescence intensities in chamber 2 (MYSY) and chamber 3 (CCYV) increased after heating for more than 40 and 30 min, respectively. These results revealed that multiple RNA viruses, extracted from diseased cucumber leaves, can be detected simultaneously within 40–60 min, without any cross-contamination between reaction microchambers, on our diagnostic device, even though RNA is hydrolytically much less stable than DNA. Moreover, the synthesis of DNA from an RNA template via reverse transcription is required during the RT-LAMP assay. In the future, we will explore the detection limit of LAMP assays on our microfluidic diagnostic devices.

## 4. Conclusions

A microfluidic device for enabling the rapid multiplex genetic diagnosis of plant viral diseases was developed. As a necessary first step for realizing such a microfluidic device, we developed an autonomous mixing and dispensing system to allow simple operations. A chaotic mixer with periodic oblique ridges exhibited a high mixing efficiency of almost 90% within a short flow distance of at least 10 mm along the flow direction. A high-precision fluid dispensing operation was made possible by controlling different burst pressures of the phaseguides with three different angles integrated into each reaction chamber, thus producing an array of chambers that could be sequentially filled with the sample with a high repeatability and reproducibility. Additionally, the visualization of LAMP assay products in the microfluidic devices could be achieved by developing a modified soft-lithography technique required to create deep localized chamber structures.

Then, we successfully demonstrated the ability of the fabricated diagnostic devices to detect a DNA-based plant virus infecting tomato, using the LAMP method. Moreover, we explored the possibility of the simultaneous detection of RNA-based plant viruses infecting cucurbits by using the RT-LAMP method. As a result, multiple RNA viruses extracted from diseased cucumber leaves were successfully detected within 60 min, without any cross-contamination between the various reaction chambers, requiring a sample/reagent volume of 3.1 µL in each of our proposed diagnostic devices. In principle, the microfluidic device has the potential to enable the multiplex genetic diagnosis of a variety of infectious diseases caused by different types of pathogens (viruses, bacteria, fungi, and parasites), not only for plants, but also for animals and humans, multiplex allergen testing in food production, and the multiplex identification of poisonous plants and illegal drugs, based on their specific genetic information. Additionally, it will allow us to perform on-site diagnostic testing without expert knowledge and skills owing to its unique abilities, such as its rapid detection, portability, simplicity, and low cost. In particular, the microfluidic device will provide a multiplex LAMP platform with an extremely simple device structure for autonomous sample mixing and dispensing, with just one operation for introducing a sample into the device.

## Supplementary Materials

The following are available online at https://www.mdpi.com/2072-666X/11/6/540/s1, Section S1: Fabrication process of the microfluidic diagnostic device; Section S2: Optimization of the detection conditions; Section S3: Fluorescence imaging in LAMP assays with a smartphone; Section S4: Detection of RNA-based plant viruses (cucumber mosaic virus (CMV) and kyuri green mottle mosaic virus (KGMMV)); Table S1: Photolithography conditions for SU-8 patterning; Table S2: Specification of fluorescence filter cubes used for selecting excitation wavelength ranges; Table S3: The average fluorescence intensities (after 60 min) either in the positive chambers (Nos. 2 and 4) or the negative chambers (Nos. 1, 3, and 5), and the intensity ratio (P/N) of negative (N) to positive (P); Figure S1: A comparison of the fluorescence intensity of the six different combinations of the two berberine compounds and three optical filter cubes (i.e., three different excitation wavelength ranges) estimated before and after the LAMP assay for 30 and 60 min in the microfluidic diagnostic devices; Figure S2: Homemade equipment for smartphone-based fluorescence imaging during multiplex LAMP assays in microfluidic diagnostic devices; Figure S3: Fluorescence images were taken with a smartphone after the LAMP assay (60 min) with berberine chloride and berberine sulfate. The positive reaction chambers (Nos. 2 and 4) are denoted by arrows in the photographs. The fluorescence intensity ratios (P/N) are also presented in the figure; Figure S4: Changes in fluorescence intensities as a function of the reaction time showing the RT-LAMP detection of an RNA-based plant virus (cucumber mosaic virus, CMV). The concentration of total RNA, containing the CMV viral RNA target, was 17.6 ng/µL after mixing with the LAMP reagents; Figure S5: Changes in fluorescence intensities as a function of the reaction time. RT-LAMP was used to detect an RNA-based plant virus (kyuri green mottle mosaic virus, KGMMV). The concentrations of total RNA, containing the KGMMV viral RNA target, were (a) 19.0 and (b) 3.0 ng/µL after mixing with the LAMP reagents. Video S1: Autonomous sample dispensing into an array of microchambers.
