# Supplementary material for: A Microfluidic Diagnostic Device Capable of Autonomous Sample Mixing and Dispensing for the Simultaneous Genetic Detection of Multiple Plant Viruses
Source: Micromachines (Basel). 2020 May 26;11(6):540. doi: 10.3390/mi11060540 (PMC7344993; doi:10.3390/mi11060540)
Supplement: Supplementary file 1 [file micromachines-11-00540-s001.zip › micromachines-808996 proof done/Supplementary Materials.docx]

Supplementary Materials

A Microfluidic Diagnostic Device Capable of Autonomous Sample Mixing and Dispensing for the Simultaneous Genetic Detection of Multiple Plant Viruses

Daigo Natsuhara, Keisuke Takishita, Kisuke Tanaka, Azusa Kage, Ryoji Suzuki,
Yuko Mizukami, Norikuni Saka, Moeto Nagai and Takayuki Shibata

S1. Fabrication Process of the Microfluidic Diagnostic Device

The fabrication process of polydimethylsiloxane (PDMS)-based microfluidic devices by using a modified soft-lithography process assisted by hemispherical polymer beads is as follows.

(a) Wafer Cleaning

As a starting material, single-crystal silicon wafers (e-Prize Co., Yokohama, Japan) were immersed in a 4:1 mixture of H_2_O_2_ and H_2_SO_4_ at a temperature above its boiling point for 10 min, followed by rinsing and blow-drying with deionized (DI) water and nitrogen gas, respectively.

(b) Photolithography for Patterning of the SU-8 Mold

A negative thick photoresist (SU-8 3050, MicroChem Corp., Newton, MA, USA) was used for the fabrication of an SU-8 master mold pattern. The SU-8 mold consisting of an array of five microchambers and a microchannel network connected to them were patterned on a Si wafer (after cleaning as described above) by a two-step photolithography process. The photolithography conditions for SU-8 patterning are detailed in Table S1. The first layer of the SU-8 pattern was formed for an array of microchambers connected to a microchannel network. The thickness was adjusted to approximately 40 µm. The second layer of the SU-8 pattern was formed by aligning it to the first layer for ridge structures integrated into the microchannel, which were used for a chaotic mixer and phaseguides for autonomous sample mixing and dispensing, respectively. The thickness was adjusted to approximately 40 µm. The heights of the ridge structures and the microchannel were adjusted to be approximately 40 and 80 µm, respectively.

(c) Modified Soft-Lithography Process Supported by Hemispherical Polymer Beads

To create deep localized microchamber structures, hemispherical polymer beads (2 mm in diameter, SAYAKOBO, Yokohama, Japan) were glued at a center position on the top surface of each SU-8 chamber pattern with epoxy adhesive (Araldite, Huntsman Japan, Kobe, Japan) at room temperature for 12 h. Then, the SU-8 master mold was replicated in polydimethylsiloxane (PDMS, Silpot 184, Dow Corning Toray Co., Ltd., Tokyo, Japan) after curing at 80 °C for 40 min on a hot plate (EC1200-N, AS ONE, Osaka, Japan). Finally, after peeling the sample off the SU-8 master, circular holes (1.0 mm in diameter) for the inlet and outlet ports were punched into the PDMS microfluidic devices by using a biopsy punch piercing tool (Kai Industries Co. Ltd., Gifu, Japan). It should be noted that PDMS was premixed with a curing agent at a ratio of 10:1, followed by stirring and degassing under reduced pressure for 3 min with mixing equipment (V-mini300, EME Corp., Tokyo, Japan). Then, the PDMS mixture was poured onto the SU-8 master and degassed again at ~70 kPa for a few minutes before curing.

**Table S1.** Photolithography conditions for SU-8 patterning.

|  | **1st Layer** | **2nd Layer** |
| --- | --- | --- |
| **Thickness** | ca. 40 µm | ca. 40 µm |
| **Spin coating** ^a)^ | Slope (5 s) → 500 rpm (10 s) → Slope (10 s)  → 3000 rpm (30 s) for 1^st^ layer or 4000 rpm (30 s) for 2^nd^ layer  → Slope (10 s) | |
| **Pre-baking** ^b)^ | 65 °C (2 min) → 95 °C (15 min) → 65 °C (2 min) → Room temp. (5 min) | |
| **UV exposure** ^c)^ | 250 mJ/cm^2^ | |
| **Post-baking** ^b)^ | 65 °C (2 min) → 95 °C (5 min) → 65 °C (2 min) → Room temp. (5 min) | |
| **Development** ^d)^ | ― | 10 min (Room temp.) |
| **Rinsing** ^e)^ **and Drying** ^f)^ | ― | (as appropriate) |

^a)^ Spin coater (1H-DX2, Mikasa Co., Ltd., Tokyo, Japan); ^b)^ hot plate (EC-1200N, AS ONE, Osaka, Japan); ^c)^ mask aligner (PEM-800, Union Optical Co., Ltd., Tokyo, Japan); ^d)^ developed with 2-methoxy-1-methylethyl acetate (FUJIFILM Wako Pure Chemical Corp., Osaka, Japan); ^e)^ rinsed with acetone (FUJIFILM Wako Pure Chemical Corp., Osaka, Japan), and then isopropanol (Kanto Chemical Co., Inc., Tokyo, Japan); ^f)^ blow-drying with nitrogen gas.

S2. Optimization of the Detection Conditions

As a preliminary experiment, we first optimized the detection conditions of target DNA amplified by the loop-mediated isothermal amplification (LAMP) method, that is, an ideal combination of DNA-binding fluorescent dyes and the appropriate excitation wavelength for each dye. In the experiments, two types of berberine compounds, consisting of berberine chloride hydrate (FUJIFILM Wako Pure Chemical Corp., Osaka, Japan) and berberine sulfate hydrate (Tokyo Chemical Industry Co., Ltd., Tokyo, Japan), were chosen as fluorescent indicators in the LAMP assay. Additionally, the appropriate excitation wavelength was investigated under irradiation selected from a mercury light source (C-SHG1 Super High Pressure Mercury Lamp Power Supply, Nikon, Tokyo, Japan) with three optical filter cubes (UV-2A, BV-2A, and B-2A, Nikon, Tokyo, Japan). The specifications of the optical filter cubes are listed in Table S2.

Figure S1 shows a comparison of the fluorescence intensity of the six different combinations of the two berberine compounds and three optical filter cubes (i.e., three different excitation wavelength ranges) estimated before and after the LAMP assay for 30 and 60 min in the microfluidic diagnostic devices. The DNA-based plant virus, that is, tomato yellow leaf curl virus (TYLCV) Israel strain, was detected in the experiments. A primer set for the detection of TYLCV was pre-spotted and dried in reaction chambers (Nos. 2 and 4; denoted by symbol *P* in the graph), while distilled water (DW) was pre-spotted in the other reaction chambers (Nos. 1, 3, and 5 ; denoted by symbol *N* in the graph) as a negative control. All of the experimental results showed that the fluorescence intensities of reaction chambers containing the primer set (Nos. 2 and 4) were higher than those of the other chambers (Nos. 1, 3, and 5 as a negative control). Among these combinations, however, the strongest fluorescence intensities in both the berberine compounds could be observed using an optical filter BV-2A (blue-violet light irradiation at 400–440 nm) compared to UV-2A (UV light irradiation at 330–380 nm) and B-2A (blue light irradiation at 450–490 nm).

The average fluorescence intensities (reaction time: 60 min) obtained from the positive chambers (Nos. 2 and 4) or the negative chambers (Nos. 1, 3, and 5), and the intensity ratio (P/N) of negative (N) to positive (P), are summarized in Table S3. Although the fluorescence intensity ratio (P/N) obtained with berberine chloride was slightly higher than that with berberine sulfate, the negative fluorescence intensity (N) obtained with berberine chloride was lower than that with berberine sulfate, resulting in a higher contrast of positive (P) to negative (N). As a result, a combination of berberine chloride and an optical filter BV-2A was determined to be the most appropriate for the LAMP assay in microfluidic diagnostic devices.

**Table S2.** Specification of fluorescence filter cubes used for selecting excitation wavelength ranges.

| **Fluorescence  Filter Cubes** | **Excitation Filter (nm)  (Bandpass Filter)** | **Dichroic Mirror (nm)  (Longpass Filter)** | **Barrier Filter (nm)  (Longpass Filter)** |
| --- | --- | --- | --- |
| **UV-2A** | 355/50  (330–380) | 400 | 410 |
| **BV-2A** | 420/40  (400–440) | 455 | 460 |
| **B-2A** | 470/40  (450–490) | 505 | 510 |


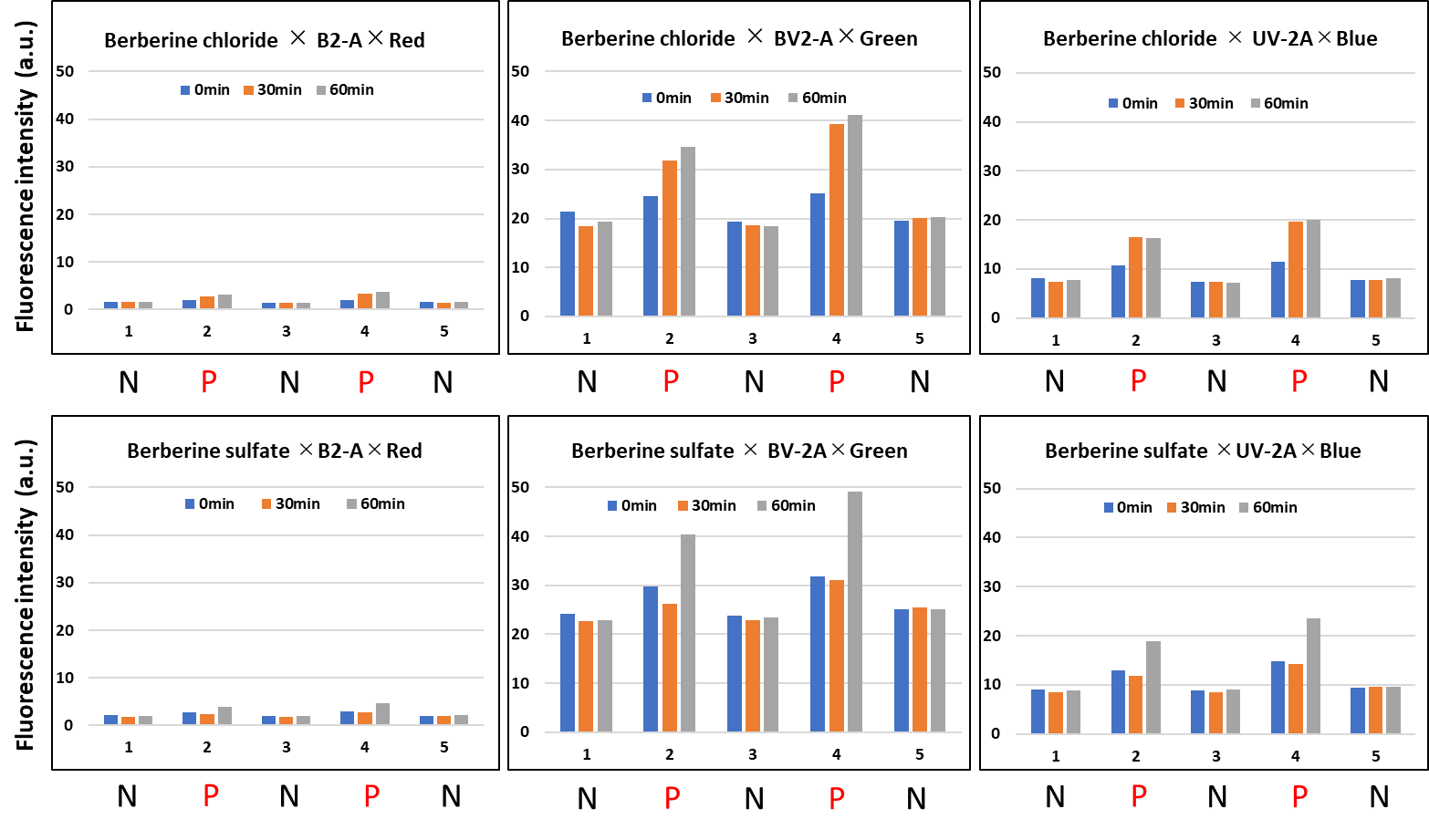


**Figure S1.** A comparison of the fluorescence intensity of the six different combinations of the two berberine compounds and three optical filter cubes (i.e., three different excitation wavelength ranges) estimated before and after the LAMP assay for 30 and 60 min in the microfluidic diagnostic devices.

**Table S3.** The average fluorescence intensities (after 60 min) either in the positive chambers (Nos. 2 and 4) or the negative chambers (Nos. 1, 3, and 5), and the intensity ratio (P/N) of negative (N) to positive (P).

| **Fluorescence Filter Cubes** | **Berberine Chloride (180 µM)** | | |
| --- | --- | --- | --- |
|  | **Negative (N)** | **Positive (P)** | **Ratio (P/N)** |
| **UV-2A** | 7.67 | 18.14 | 2.36 |
| **BV-2A** | 19.31 | 37.91 | 1.96 |
| **B-2A** | 1.59 | 3.41 | 2.14 |
| **Fluorescence Filter Cubes** | **Berberine Sulfate** **(180 µM)** | | |
|  | **Negative (N)** | **Positive (P)** | **Ratio (P/N)** |
| **UV-2A** | 9.17 | 21.15 | 2.31 |
| **BV-2A** | 23.78 | 44.79 | 1.88 |
| **B-2A** | 2.04 | 4.34 | 2.13 |

S3. Fluorescence Imaging in LAMP Assays with a Smartphone

To simplify the fluorescence imaging in LAMP assays, we demonstrated that a fluorescence microscope can be replaced by a smartphone modified with homemade equipment, as shown in Figure S2. An array of LEDs (peak wavelength: 445 nm), after passing through an optical shortpass filter (cutoff wavelength: 475 nm), was used as a light source, and illuminated the microfluidic diagnostic devices immersed in a hot-water bath at 63 °C. Fluorescence observations during the LAMP assay could be recorded with a smartphone via an optical longpass filter (cutoff wavelength: 530 nm), which was positioned on the top surface of a dark box (470 mm × 450 mm × 270 mm).

Figure S3 shows the fluorescence images taken with a smartphone during the LAMP assays (at 60 min) for the detection of DNA-based virus (TYLCV) with berberine chloride and berberine sulfate as fluorescent indicators. The fluorescence contrast of negative chambers (Nos. 1, 3, and 5) to positive chambers (Nos. 2 and 4; denoted by an arrow) with berberine chloride was better than berberine sulfate because of the lower fluorescence intensity observed in the negative control with berberine chloride. These results are consistent with those obtained with the fluorescence microscope described above. It should be noted again that the visualization of LAMP assays with a smartphone will make multiplex diagnosis easier on microfluidic devices.


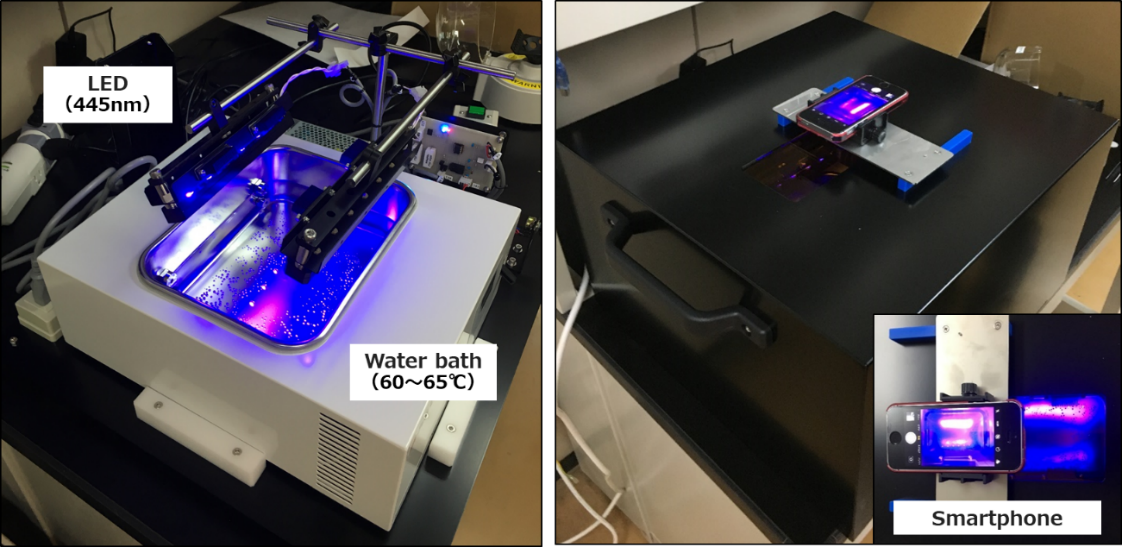


**Figure S2.** Homemade equipment for smartphone-based fluorescence imaging during multiplex LAMP assays in microfluidic diagnostic devices.


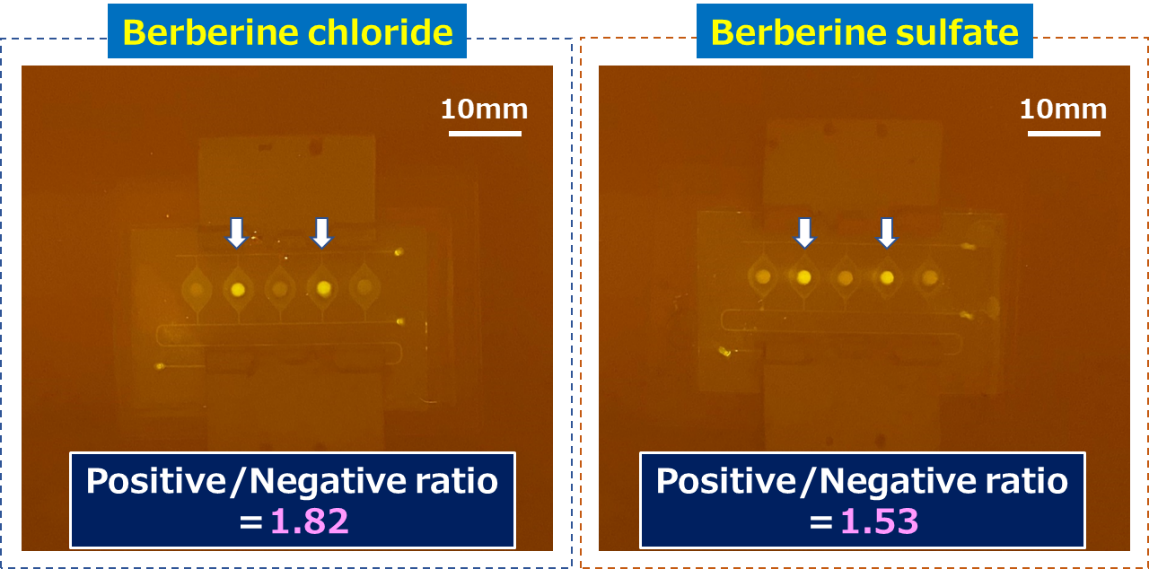


**Figure S3.** Fluorescence images were taken with a smartphone after the LAMP assay (60 min) with berberine chloride and berberine sulfate. The positive reaction chambers (Nos. 2 and 4) are denoted by arrows in the photographs. The fluorescence intensity ratios (P/N) are also presented in the figure.

S4. Detection of RNA-Based Plant Viruses (CMV and KGMMV)

Figure S4 shows the results of the detection of CMV (viral RNA) by the RT-LAMP assay on the fabricated microfluidic device. Firstly, the total RNA containing the CMV viral RNA target, extracted from infected celery leaves, was mixed with LAMP reagents to a concentration of 17.6 ng/µL and introduced into a microfluidic device. As expected, the fluorescence intensity only markedly increased in chamber 5 (CMV) after heating for more than 20 min.

Next, total RNA extracted from cucumber leaves infected with KGMMV was mixed with LAMP reagents to a concentration of 19.0 ng/µL and loaded into a microfluidic device. This assay gave a false positive amplification in chamber 3, in which a primer set for the detection of CCYV had been pre-spotted. Chamber 4, with a primer set for KGMMV, showed positive amplification, as expected (see Figure S5a). However, when the concentration of total RNA was decreased to 3.0 ng/µL, false positive amplification was not seen (see Figure S5b). These results indicate that optimization of the viral RNA target concentration in a sample is particularly important to ensure a successful LAMP reaction.


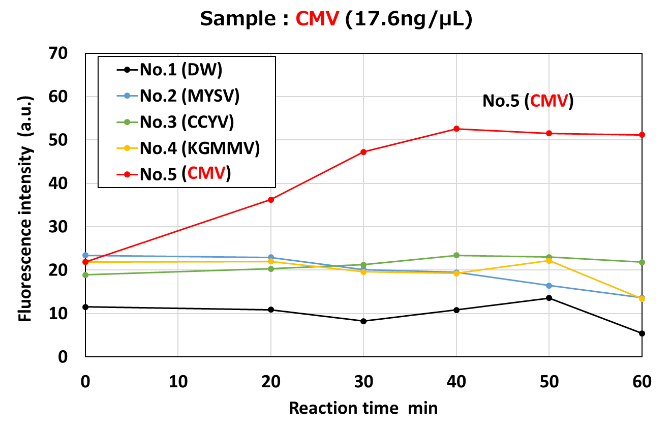


**Figure S4.** Changes in fluorescence intensities as a function of the reaction time showing the RT-LAMP detection of an RNA-based plant virus (cucumber mosaic virus, CMV). The concentration of total RNA, containing the CMV viral RNA target, was 17.6 ng/µL after mixing with the LAMP reagents.


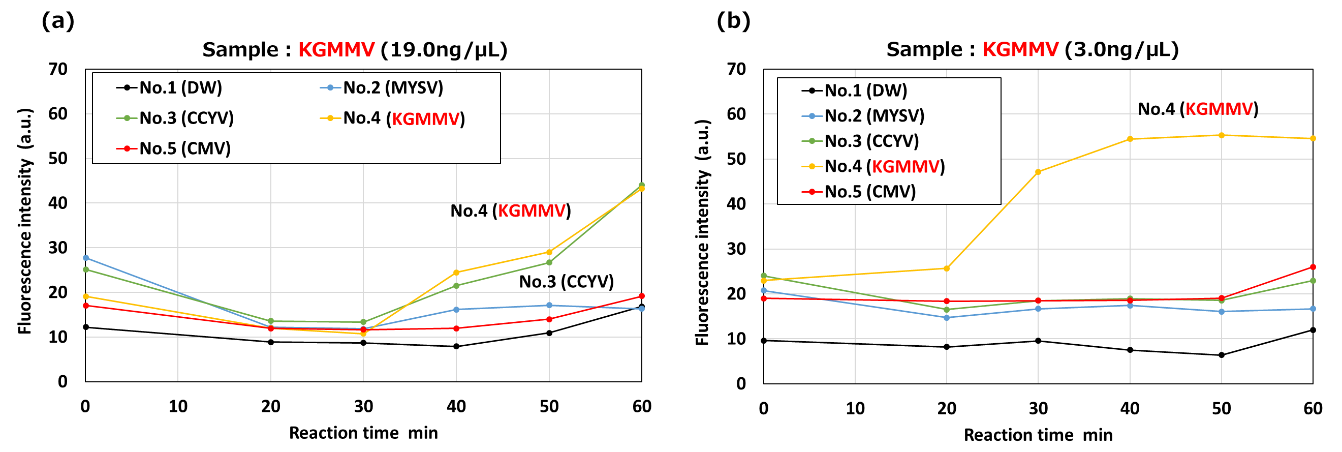


**Figure S5.** Changes in fluorescence intensities as a function of the reaction time. RT-LAMP was used to detect an RNA-based plant virus (kyuri green mottle mosaic virus, KGMMV). The concentrations of total RNA, containing the KGMMV viral RNA target, were (**a**) 19.0 and (**b**) 3.0 ng/µL after mixing with the LAMP reagents.

| 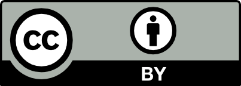 | © 2020 by the authors. Submitted for possible open access publication under the terms and conditions of the Creative Commons Attribution (CC BY) license (http://creativecommons.org/licenses/by/4.0/). |
| --- | --- |
